# Revisiting water resources management in the Mandara Mountains

**DOI:** 10.1016/j.heliyon.2025.e41692

**Published:** 2025-01-03

**Authors:** Diane Madomguia, Esther Laurentine Nya, Emma Laureane Njomou-Ngounou, Nadège Gatcha-Bandjun, Tulinave Burton Mwamila, Jules Balna, Emina Halimassia, Jules Metsebo, Wilfried Arsène Letah Nzouebet, Raoul Rodrigue Tchoumbe, André Firmin Bon, Willis Gwenzi, Chicgoua Noubactep

**Affiliations:** a Department of Hydraulics and Water Management, National Advanced School of Engineering, University of Maroua, P.O. Box 58, Maroua, Cameroon; b Faculty of Arts, Letters and Social Sciences University of Bertoua, P.O. Box: 416, Bertoua, Cameroon; c Eau Potable Pour Tous (EPPT), P.O. Box 9362, Bangangté, Cameroon; d Department of Geography, University of Yaoundé I, Yaoundé, P.O. Box 755, Cameroon; e Department of Applied Chemistry, Faculty of Science, Box 118, University of Ebolowa, Cameroon; f Department of Applied Chemistry, Faculty of Science, Box 46, University of Maroua, Cameroon; g Water Institute, Ministry of Water, Dar es Salaam, P.O. Box 35059, Tanzania; h NTWAM Water & Environment Initiative, P.O. Box 1041, Dar es Salaam, Tanzania; i Faculty of Arts, Letters and Social Sciences, University of Maroua, Maroua, P.O. Box 644, Cameroon; j Faculty of Science and Technology, Campus of Banekane, Université des Montagnes, P.O. Box 208, Bangangté, Cameroon; k Department of Meteorology and Climatology, National Advanced School of Engineering, University of Maroua, P.O. Box 58, Maroua, Cameroon; l Department of Basic and Applied Fundamental Sciences, Higher Institute of Agriculture, Forestry, Water and Environment, Box 118, University of Ebolowa, Cameroon; m Biosystems and Environmental Engineering Research Group, 380 Adylin, Westgate, Harare, Zimbabwe; n Centre for Modern Indian Studies (CeMIS), University of Göttingen, Waldweg 26, D-37073, Göttingen, Germany; o Department of Applied Geology, University of Göttingen, Goldschmidtstraße 3, D-37077, Göttingen, Germany

**Keywords:** Annual flooding, Integrated water resources management, Kilimanjaro concept, Landscape restoration, Mandara mountains, Rainwater harvesting, Soil water preservation

## Abstract

This article evaluates the prospects for rainwater harvesting (RWH) as a means of optimizing water management in the Mandara Mountains. RWH is a small-scale water conservation approach for locally intercepting and storing rainfall before it enters the usual hydrologic cycle. This ancient practice has recently sustained lives in semiarid areas of the world (e.g. Brazil, China), but is not yet really used in the Mandara Mountains of Cameroon where people are still lacking safe drinking water. Recently, RWH was also demonstrated as the missing puzzle in integrated water resources management (IWRM) not only in areas facing water scarcity. The present article aims to prepare large scale RWH in the Mandara Mountains. Water harvesting yields are estimated for residential roofs, administrative and confessional buildings, and agricultural areas (e.g. farm scale). The results show that RWH is an affordable, applicable, and attractive tool for both rural and urban communities to sustainably solve the long-lasting problem of lack of safe drinking water in the Mandara Mountains. Moreover, despite the short rainy season, RWH may provide enough irrigation water to mitigate dry spells and increase agricultural and livestock productivity. This study is regarded as a blueprint for planning sustainable water management in the Mandara Mountains.

## 1 Introduction

The world water demand is escalating because of accelerated industrialization, accelerated urbanization, climate change, growing population, and growing energy demands [[1], [2], [3], [4], [5], [6]]. Therefore, it is urgent to explore and implement effective strategies that surpass conventional methods of freshwater supply (Table 1) [[7], [8], [9], [10], [11], [12], [13], [14]]. Past and current efforts to address water scarcity in various regions facing recurrent water crises are based on a rather pragmatic management approach resulting from the European sanitation revolution of the 19th century [8,[15], [16], [17], [18], [19]]. A key feature of this approach is that rainwater has been considered as a waste for more than a century (1850–1980). The European approach of water management (supply and sanitation) is considered conventional, everything else is alternative [18,19]. A recent overview article by Loper et al. [20] excellently summarizes this view by recalling that alternative water sources are sustainable sources of water, not coming from surface water and groundwater. For these authors alternative water sources include harvested rainwater and reclaimed wastewater. The present article, focusing on the Mandara Mountains of Cameroon, aims to show that the view of Loper et al. [20] is not intuitive. In particular, rainwater and wastewater cannot be given the same weightage.

**Table 1:** Selected arguments rationalizing the barriers compromising safe drinking water supply in resource-constrained settings. IWRM stands for Integrated water resources management.

Variable	Corresponding limitations	Countermeasures
Climate	Semi-arid and hilly regions are prone to water shortages	Boreholes, RWH
Climate change	Decreasing availability and declining quality	IWRM
Funding	Centralized systems are expensive and difficult to operate	Appropriate technologies
Natural disasters	Poor countries are less prepared to face disasters	International relief
Political stability	There are wars and unstable conditions in many locations	International missions
Population	Water is a finite resource, but the demand is growing	Expand supply chain
Scholar education	Non educated people are difficult to sensitize	Appropriate training tools
Technology	Centralized systems are expensive, alternatives are scarcy	Frugal technologies
Urbanisation	No piped water in poorly planned urban areas	Decentralized options

The European paradigm of water management is rooted in urban water infrastructures of underground piping networks for water distribution and sewer collection [7,9,13,16,17,21,22]. The design of sewer drainage systems is challenging because it is difficult to predict the volume of rainwater to drain to the river [23]. The European society is now aware of the vulnerability of these century-old centralized water management strategies and their incapacity to meet future environmental and societal challenges [8,[10], [11], [12],^22^,^24^,^25^]. Additionally, centralized water management systems are reaching the end of their design lifetimes [9,14,22,[26], [27], [28]]. The question arises how to replace the related infrastructures while simultaneously coping with high water quality standards? During the past three to four decades some scientists have been questioning the suitability of centralized systems in developed countries [8,12,24,25,[29], [30], [31], [32], [33], [34]]. Large-scale rainwater harvesting has then been introduced as a powerful tool to save piped drinking water [13,22]. Surprisingly, this discussion co-exist in the literature with views that developing countries have limited capital for the installation of centralized water management infrastructures (Table 1) [9,12,31]. In other words, there is a generalized refusal to acknowledge that rainwater is freshwater and the same can be a recyclable first choice for water supply (for all uses). The present article posits that this state of affairs justifies the poor situation of water management in the Mandara Mountains of Cameroon. Here also, people navigate with misconceptions (e.g. acid rain) and legends (e.g. rainmaking) about rainwater [35], while waiting for the (promised) centralized piped water from the state [[36], [37], [38], [39]].

Efforts to promote or revitalize RWH in the Mandara Mountains of Cameroon can be traced back to the end of the 1970s and the early 1980s [[40], [41], [42], [43], [44], [45], [46], [47], [48]]. In fact, Zalla et al. [44] reported that, in the mid-1970's the Government of Cameroon adjusted its development strategy in the then "Northern Province" to the amelioration of mountain life. This strategy was mirrored in its fourth Five-Year Plan (1976–1981) and entailed on "identifying potential interventions which can increase agricultural and livestock production, monetary income, and the welfare of rural people over the next five to ten years" (from 1981 on). Within this framework, development activities responding to the water needs of the Mandara people involved the construction of 57 small dams [49,50]. The small dams were partly inspired by an indigenous system locally called biefs [41,44,[51], [52], [53], [54], [55]]. Despite the small dams and subsequent efforts, the problem of water scarcity in the Mandara Mountains is still largely unresolved [[56], [57], [58], [59], [60], [61], [62]]. Accordingly, there is an urgent need to address this issue while considering recent progresses on implementing integrated water resource management [18,19,26,[63], [64], [65], [66]].

Damien Clément carried out extensive studies on technical choices for water supply in Mandara Mountains of Cameroon from 1960 to 1990 [48,67]. The State of Cameroon and foreign actors (experts) have been trying to obtain drinking water for mountain dwellers during the long dry season (8–9 months). Through programs for village water supply called PHV (PHV = Programmes d'Hydraulique Villageoise). With the PHV programs, Western experts intervened regularly in: (i) creating new water points (i.e. boreholes and small dams), and (ii) deepening of existing wells. In other words, the Western expertise has used costly sophisticated techniques to “mine” existing water from a deeper ground. In the perspective of water management, techniques were introduced to contribute to the depletion of underground reservoirs, without any effort to recharge the aquifer [18,68]. Clément [67] pointed out that although the used approaches have generally improved water supply in the Mandara, it does not mean that the named techniques are adapted to solving the problem. In fact, a more general issue has not been discussed: the choice of the strategy to obtain water in the Mandara Mountains. Instead of the “mining strategy” towards “water production” (borehole, dam, well), Clément [48,67] proposed the “renewal strategy” as an alternative. The renewal strategy involves improving the availability of water for mountain dwellers. It is based on the regeneration of existing aquifer reserves though controlled infiltration, rather than the creation of new water points and the search for hypothetical productive aquifers. This option is not new in the Mandara Mountains [35,69]. On the other hand, it corresponds to the old view that “No drop of water should flow into the sea without serving the interest of man” (King Parakramabahu of Sri Lanka - 12th century). In other words, the renewal strategy has been neglected for the past 70 years in the Mandara Mountains [37,38,48,67].

Clément's works [48,67] have demonstrated that the “renewal” strategy has been confirmed for centuries. Consequently, the choice of the “mining” option was made against a plausible, yet endogenic strategy, and not in absolute terms. The “renewal” strategy implies maximizing infiltration of rainwater to make springs and wells more productive during the dry season, and to decrease the severity of flooding during the rainy season [68,70,71]. Progresses in civil engineering have the capacity to expand water retention in the Mandara Mountains to the extent that there are no more “powerful torrents” to be drained by the temporal rivers (mayos) or no more flooding, despite heaving rainfalls. In other words, there is room for “zero torrent” or “zero runoff” from the Mandara Mountains [18,19,72]. It is expected that rainwater harvesting will soon transform the whole Mandara Mountains from a region of extreme water scarcity to a domain where self-sufficient clean drinking water supply is a reality.

The objective of this work is to present a concept rooting integrated water resource management (IWRM) in rainwater harvesting (RWH) at the smallest scale (farm, household) and demonstrate its applicability in the Mandara Mountains as a case study. It is about a realizable holistic approach incorporating household-scale and farm-scale decentralized RWH systems into IWRM both in rural and urban environments. This framework provides a solid foundation for a paradigm shift toward mitigating water insecurity or improving water security in the Mandara Mountains. Clearly, it is about raising awareness on maximizing the development and use of all locally available water resources including rainwater and stormwater runoff, for local consumption. Furthermore, food security is an important issue in the Mandara Mountains. Coupling food production and decentralized water management is a viable solution to support sustainable living initiatives and community development in these hilly areas.

This research analyzes key factors and the mutual relationships important for establishing RWH as a first choice for water supply in the Mandara Mountains. It capitalizes on residential scale RWH, which was hardly mentioned in early theorizing efforts [37,38,59] and not mentioned during the baseline investigations of the 1970s [48,67]. Residential scale RWH currently has a prominent place in the literature [14,25,27,28] and in discussions on local development at the community level [18,19].

The presentation starts with the methodology used for the literature review (Section 2), followed with a description of the Mandara Mountains and its water harvesting potential (Section 3). Section 4 gives an overview of past and current perceptions of the potential of RWH to address water supply in this hilly region. Section 5 conceptualizes the future of water management rooted in RWH, and Section 6 discusses its realization, while outlining some aspects of how the new approach will increase the living standards in the Mandara Mountains. A short conclusion (Section 7) closes the presentation.

## 2 Methodology

An appraisal approach is used herein, consisting in collating the huge scientific literature on rainwater harvesting in the Mandara Mountains and extracting points of certainty and uncertainty, knowledge gaps and open questions. An appraisal does identify levers for action [73,74]. The review is based on the authors’ expert knowledge. For this purpose, an authorship with skills in the following disciplines were mobilized: biogeochemistry, civil engineering, ecology, environmental chemistry, environmental remediation, hydrogeochemistry, hydrology, urban geography, water engineering, water management and water quality. A large bibliographic corpus was selected from all available sources, including Google Scholar, Researchgate and Web of Science. Scientific articles validated by peers, qualification works (e.g. master, PhD), scientific reports, and technical reports were considered.

The premise is that rainfall is a valuable resource and should be harvested everywhere in the Mandara Mountains, whether it is abundant or not. This is because only a tiny fraction of rainfall is permanentaly available as groundwater (e.g. boreholes, tubewells, wells) or surface water (e.g. lakes, rivers, springs) [18,[75], [76], [77], [78], [79], [80]]. Clearly rainfall should be regarded as a stand-alone source of freshwater, capable of supplying certain communities with water for all uses. In other words, by only optionally considering RWH, the conventional approach of integrated water resources management (IWRM) is not really holistic. The methodolody used herein consists in mining the literature to falsify arguments that have been impairing the full consideration of RWH in IWRM efforts using the Mandara Mountains as case study. In other words, selected success stories in favour of a new approach rooting IWRM on RWH are presented and discussed. In this effort, a particular attention was given to cases where rainwater has been used as source of drinking water.

Quantity of rainwater harvested from a given rainfall runoff is estimated using rational method, which comprises of the following parameters, catchment size (m^2^), runoff coefficient (representing relationship between rainfall and runoff) and rainfall intensity (mm).

For illustration, the following can be considered:

Conventional IWRM: Supply = (ground + surface) water + eventually (rain + recycled) water (“groundwater and surface water first”).

RWH-based IWRM: Supply = rainwater + eventually (ground + surface + recycled) water (“rainwater first”).

The key difference is that the alternative IWRM approach gives full consideration to RWH ("rainwater first"). More so, groundwater and surface water are only optionally considered, for example where rainfall is not abundant (like in the Mandara Mountains). The key advantage of the alternative system is that erosion and flooding are addressed at the source, while water supply is hugely augmented [18,19].

## 3 The Mandara Mountains

### 3.1 General aspects

**Fig. 1:**
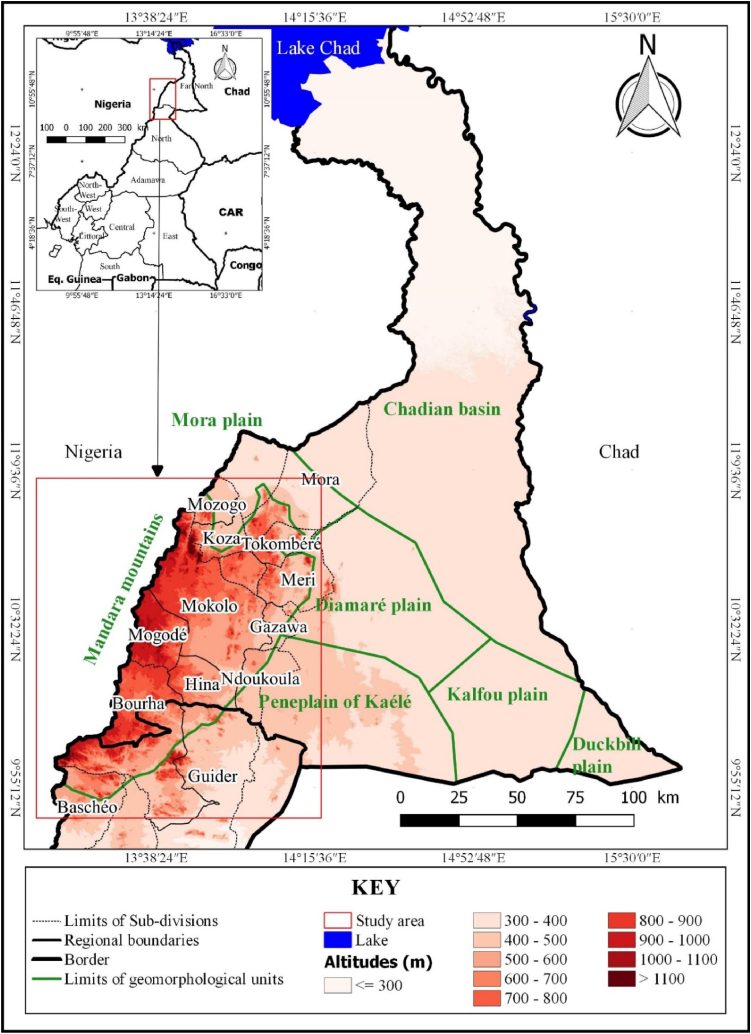
Location of the Mandara mountains in Cameroon.

The Mandara Mountains is a hilly region located in Cameroon and Nigeria (Fig. 1) [[81], [82], [83]]. The Cameroonian side is the subject of this work and corresponds administratively to: (i) the departments of Mayo Sava, Mayo Tsanaga, (ii) the Sub-division of Meri, (department of Diamaré) and Sub-division of Mayo-Oulo (department of Mayo-Louti) in the North Region, and (iii) 16.2 % of the total area of the Far North Region. Geographically, Mandara Mountains range between 10°32′ and 10°58′ North latitude and between 13° 20′ and 13° 40’ East longitude. The real extension of the study area is 7660 km^2^ [84]. The area holds a total of 1,467,396 inhabitants, according to the April 2010 report from the Cameroon Central Bureau of Population Censuses and Studies [85]. The maximum rainfall ranges between 800 and 1200 mm/year [62,86,87]. The area thus received a relavitely high amount of precipitation between June and September. However, most of that washed out as surface runoff [[88], [89], [90]]. Geomorphologically, this area comprises three types of landscape: mountainous ridges, intra-mountainous plateaux and piedmonts [[91], [92], [93], [94]]. There is no single perennial river flowing through the study area. As a result, there is high runoff during the rainy season (3–4 months) and no rain during the dry season (8–9 months). A noticeable water scarcity is reported every year [46,59,62].

### 3.2 Water scarcity

**Fig. 2:**
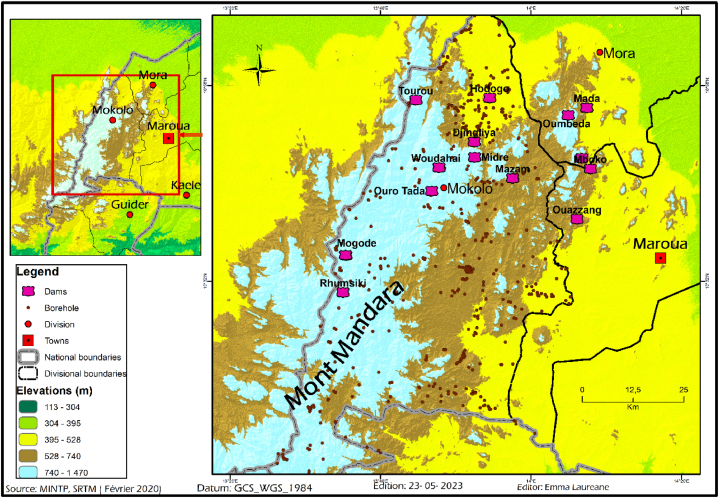
Spatial distribution of dams and boreholes in the Mandara Mountains.

The climatic and geomorphological conditions of the Mandara Mountains are at the root of the water shortage in this area, which is the rainiest in the Far North Region of Cameroon. Since the drought of 1972–1973, drinking water supply conditions have deteriorated further each year [40,42,43,61]. During the dry season, water is very scarce, because there is a general lowering of the water table due to the absence of rainfall. The daily search for water is difficult because the wells built by the mountain dwellers have dried up [37,53,54,87]. Women and children can sometimes travel up to 10 km to reach a water point. Water shortages peak in March and April. During this period of water insecurity, the high prevalence of water-borne diseases results from the consumption of contaminated water [55,[95], [96], [97], [98]]. Faced with this situation, national and international initiatives have been developed to improve the coverage of water accesibility in this area, including the construction of several dams (Fig. 2) and masonry wells [48,67]. However, most of these decentralized facilities are seasonal or run dry towards the end of dry season. This is mainly due to the high evapotranspiration. The economy is dominated by intensive and subsistence farming, large and small livestock rearing, and petty trade [61,99,100]. All these activities need water to prosper.

### 3.3 The rainwater potential of the Mandara Mountains

The quality of harvested rainwater is a major concern of RWH systems and has been well-researched over the decades [[101], [102], [103]]. It is established that with proper care and maintenance, RWH systems produce high quality, potable, soft water [18,102,104]. This study does not focus on the quality of rainwater, but rather on the feasibility of using rainfall as the primary water resource in the Mandara Mountains. Clearly, the question to answer is to which extent RWH can be considered a stand-alone tool and first choice water supply approach in this hilly region. Mountains are often considered as "water towers for humanity" [105], supplying freshwater for the adjacent lowlands.

Rain falling on any area (e.g., courtyard, farm, roof, rock) can be largely but never completely captured (harvesting efficiency lower than 100 %). This is because the runoff coefficients depend on: (i) the geometry of the roof, (ii) the roof material, and (iii) whether and to each extent the roofs are covered with water absorbing materials. On the other hand, there is always some water adsorption and evaporation on the roof surface. The amount of rainfall (Q) that can be captured from a given area (A) is estimated using Equation (1):

(1) Q = C × P × A

Where P is the average annual precipitation (mm/year) and C the coefficient of runoff of the roof catchment (C < 1, C = 1 corresponds to 100 %).

Section 3.1 recalled that 800 mm–1200 mm of rain falls on each area within the Mandara Mountains every year. Assuming that only 50 % (C = 0.5) can be harvested this corresponds to 3.06 × 10^6^ to 4.59 × 10^6^ m^3^ of water when the pluviometry varies from 800 to 1200 mm. This amount of water would supply more that 300,000 people with 25 L/day every year. Actually, this amount of water is flowing down to the lowlands where it becomes a threat in some cases.

At the residential level, the same calculation shows that an iron roof area (C = 0.9) of 100 m^2^ harvests a minimum of 72 m^3^ per year and this amount of water guarantees 25 L/day for 9 persons. 9 persons is larger than the average size of a family in the Mandara Mountains [81,82,98]. Clearly, RWH alone potentially solves the long-lasting challenge of supplying safe drinking water in this hilly population. This includes remote and scattered people in their rural areas where no centralized water supply system will reach, even in a century [82,98]. Herein, it is discussed how the use of harvested rainwater may emerge as a sustainable solution and contribute to reduce negative impacts on the environment. Interestingly, available efforts are discussing the option of harvesting rainwater during the rainy season and store it for the three driest months (April, May, June) [36,37,54].

#### 3.3.1 How much rainwater can be harvested at each scale?

At the macro-level, with an average annual rainfall of 1000 mm, if harvested within the whole Mandara Mountains of Cameroon with an efficiency of 50 %, extending over an area of 7660 km^2^, is capable of yielding 9.9 L per capita per day, making the whole area self-sufficient in drinking water supply. This is because a minimum of 7.5 L per day per person is recommended by the World Health Organization (WHO) for drinking and cooking [106,107].

At the meso-level, this amount of rainfall is capable of yielding 5000 m^3^ of water per hectare (10000 m^2^) per year or 18.8 m^3^ of water per hectare per day, if harvested with an efficiency of only 50 %. This volume of water will be used to enhance the productivity of individual crops, for example in small gardens. It is essential to point out that the calculations are limited to the volume of water that should have been lost as surface runoff, so that the rain-fed agricultural practice is not disturbed. In contrary, it is even sustained because rainwater infiltration is intensified.

At the micro-level, this amount of rainfall is capable of yielding (efficiency: 50 %) more than 750 L of water per day, throughout the year, if harvested in one standard urban compound (400 m^2^). Considering a house with a 100 m^2^ roof area within the compound, and a rainwater harvesting efficiency of 90 % for the roof, 90 m^3^ of water can be harvested every year, corresponding to 346.4 L of water. This volume of water will supply 9.8 persons with 25 L of water per day. This is enough water for a 7 person's household and some home gardening. Still at micro-level, each large rock can be regarded as a water producing unit and a corresponding infiltration structure or storage infrastructure (e.g. cistern or tank) constructed in its vicinity.

## 4 Current water management strategy in the Mandara Mountains

Many water management strategies have been suggested and implemented in the Mandara Mountains [46,47,53,54,98,108,109]. In the hills, there is often enough water but not naturally stored in the ideal place of use [98,110]. So there is need for engineered solutions to ensure safe and sustainable water for communities. Fig. 2 also reveals that existing small dams are not randomly scattered in the hills. This section summarizes the different ways to get water in the Mandara Mountains.

### 4.1 Natural springs and rivers

**Fig. 3:**
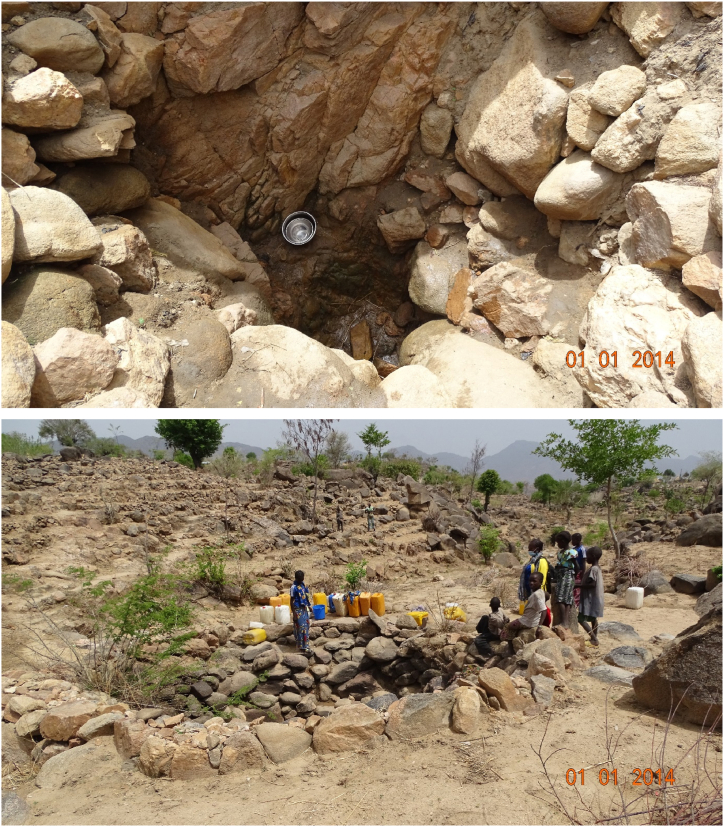
A natural spring in Djingliya (10°47′ 05″ N/13°47′ 45″ E). Toward the end of the dry season, the spring is very low in production (left), and crowded (right) because people must wait for water to accumulate (JB photo – June 2024).

Natural springs and rivers provide a reliable source for local communities. Water is collected at the source (Fig. 3) which can be some kilometers away from home. The major problem is that people must share some springs with animals [41,45,109]. That is why a protection system should be developed around each spring. The next problem of both springs and rivers in the Mandara Mountains is that they are all non perennial. This means that, in the absence of RWH infrastructures, some sort of water has to be imported into the communities for some weeks or months every year, particularly toward the end of the dry season (March to May).

### 4.2 Hand dug wells and boreholes

Hand dug well are not an appropriate method for supply in hilly regions [53,54]. This is because the water table is deep. For people living near rivers or in the plain, adopting this approach is usually the best method for water supply [47,110]. However, in the Mandara Mountains wells go dry toward the end of the dry season. For this reason, only boreholes are a real solution. However, they are very expensive and have not always been installed where people need water the most [45,62] (Fig. 2). According to two available reports from the world bank [41,45], by 1985 some 900 wells and 232 boreholes were available. It is certain that this number has increased during the past three decades. However, the water scarcity problem is far from been resolved [62]. Clearly, supplying Mandara people with groundwater is technically very challenging and financially very difficult to achieve.

### 4.3 Rainwater harvesting

Rainwater is the ultimate source of freshwater [18,101]. However, rainfall occurs in short periods of high intensity which allowing the rain falling on the surface to flow away fast [110,111]. Therefore, rainfall shall be collected and stored for later uses. Rainwater can be stored or diverted for aquifer recharge. Conventionally, rainwater is stored, everywhere there are large catchment areas (e.g. open space, roof, rock): administrative institutions, commercial premises, confessional institutions, residences, schools, and universities [59,110,112,113].

**Fig. 4:**
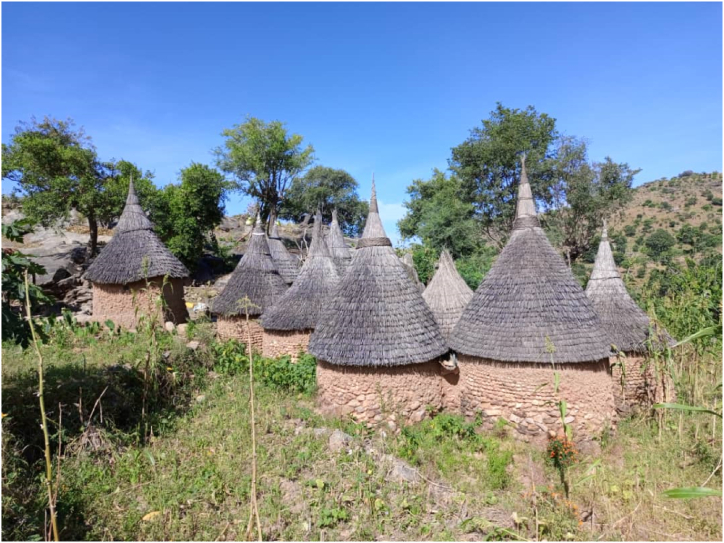
Photograph of a Mandara settlement in Magoumaz (ELN photo – Novembre 2022).

In the Mandara Mountains, Morel and colleagues have already demonstratated the technical and economical feasibility of RWH despite the small sizes of the traditional houses [46,47]. Subsequent works, summarized in Cheo [59] have claimed that the small roof sizes (Fig. 4) are a barrier to residential scale RWH in this this hilly region. Section 3.3 has recalled in tune with more recent views that this approach is faulty [18,[114], [115], [116], [117]]. In particular, ref. [114] advocated for the view that “Catching the rain at your home saves you from following the runoff down the hill”. People shall be encouraged to have larger catchment areas but they should start with what they currently have. This is because each drop of rain is important in the hills and shall be captured [114]. Moreover, water reservoirs should be scattered even in uninhabited areas, similar to retention ponds that Germany has been installling in urban areas since the 1970s [[30], [31], [32]]. Calculations (section 3) considering an average annual rainfall of 1000 mm and an efficiency of 50 %, show that on each hectare of land (10,000 m^2^), 5000 m^3^ of rainfall can be harvested and used every year. A fraction of this water can be infiltrated to sustain aquifer recharge.

### 4.4 Small dams

**Fig. 5:**
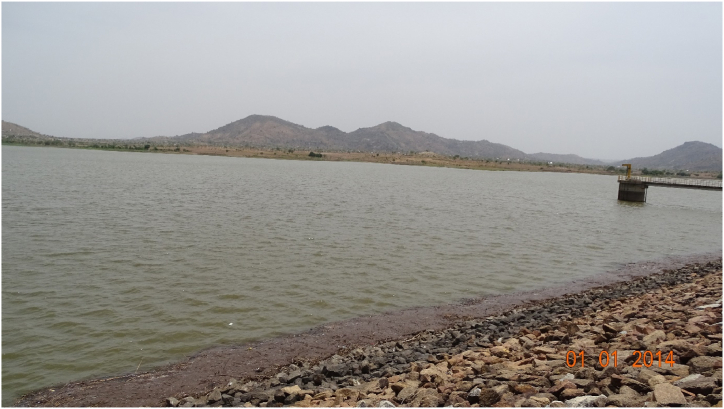
Photograph of a small dam in Douvar (JB photo – June 2024).

In hills of the whole Mandara Mountains of Cameroon, 151 potential sites for small dams were identified for the storage of springs and streams flowing out of the individual hills [41]. Of these 151 sites, 57 were priotized for construction [49], 10 were planned but only four were effectively constructed [45]. Although these water sources are ephemeral and their flows restricted to some weeks per year, storing them can help to provide communities with water for non-potable uses (e.g. gardening, livetock production). Whether some small dams were constructed after 1985 or not could not be assessed in the framework of this article, however, the still prevailing water scarcity demonstrates the untapped potential of small dams in the Mandara highlands. Fig. 5 shows a photograh of the Douvar dam, Mokolo. The calculations in section 3 have shown that several small tanks should be built at specific distances from each other. Similar to retention ponds in Germany [30] these tanks will have the double function of avoiding erosion and keeping water on the mountains. A positive side effect will be avoiding or mitigating flooding in the valleys [18,19]. It is important to empty such tanks toward the end of the dry season to keep them ready for capturing the first rains. At this stage, there is a need for some supervision. This is because such systems need some maintenance and monitoring. Monitoring is also needed to avoid the small tanks or dams from getting filled with sediments. In a cascade of tanks overflows from upper tanks are channelized to lower ones, similar to the ancient Sri Lankan cascade system [111,118,119].

## 5 New era for water management in the Mandara Mountains

The calculations made in section 3 give the amount of water that is currently not or only marginally exploited in the Mandara Mountains of Cameroon. However, they show clearly that RWH can elegantly solve the drinking water supply challenge. A challenge that has not been resolved since the 1960s despite numerous national and regional initiative, partly supported by international expertise [120] and money [67]. To complete this picture, it must be added that RWH sustains groundwater recharge and thus the productivity of local boreholes, springs and wells [18,19,113,121]. Clearly, harvesting rainwater augments water resources in the Mandara Mountains, improves its quality by avoiding pollution, while making water management everybody's business [18]. To realize this vision, this section proposes a roadmap that can be applied separately at residential and farm scales.

### 5.1 Residential scale RWH

Collecting rainwater from roofs into specially designed reservoirs mounted above the ground or in underground storage tanks is an old practice in the Mandara Mountains, particularly for houses with tin roofs [46,47,53,54,[56], [57], [58], [59]]. Although the technological feasibility and the economical viability of RWH from traditional houses (Fig. 4) has been demonstrated in the 1980s [46,47], recent works are still limiting residential RWH to modern houses with tin roofs [53,54,[56], [57], [58], [59]]. Siphambe et al. [18] argued that the cost effectiveness or the economical viability should not be regarded as important criterion anymore. This is because the volume harvested is a contribution to: (i) aquifer recharge, (ii) drinking water supply, and (iii) erosion and flood mitigation. In other words, RWH should be regarded as a decentralized and equitable water supply solution for all social groups within the Mandara Mountains. This argument alone reveals that the installation of RWH infrastructures is not a short term social responsibility benifiting the home owner, but rather a governmental issue to sustain a viable ecosystem in the long term [18,19].

The originality of the approach proposed herein is that a household is regarded as a water production unit [18,19] and no more as a potential water consumer. This perspective eases the attribution of governmental subsidies and all other forms of financial or material assistance for residential RWH infrastructures. This argument is universally valid but is crucial under hilly and semi-arid conditions where water resource is very limited. As discussed above not only the roof areas should be used for RWH but the whole compound, including courtyards and gardens. Accordingly, each residence should be equipped with both storage and infiltration infrastructures [18,19]. This task can start immediately with infiltration ponds and sand dams that are affordable because they need no (large) money expense [[122], [123], [124]]. The next step can be traditional storage pits in which infiltration pits are made impermeable with indigenous knowledge first [19].

### 5.2 Farm scale RWH

**Fig. 6:**
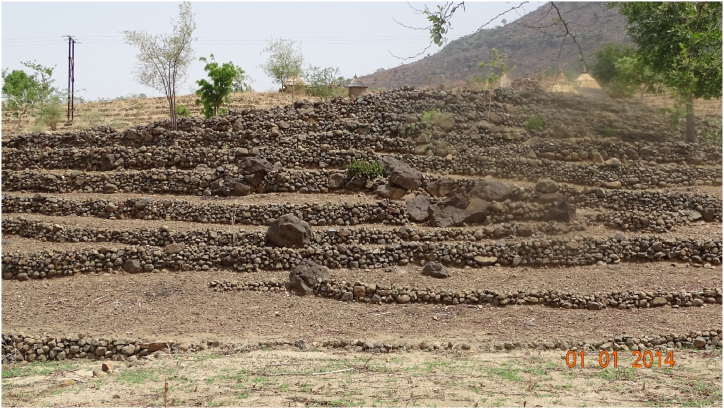
Photograph of a stone terraced system in Mouhour/Mafa (10°39′ 13″ N/13°52′ 38″ E). (JB photo – June 2024).

Terrace cultivation is the conventional approach of growing crops on sides of hills by building graduated terraces into the slope [125,126]. This method has been employed effectively for centuries to maximize arable land area and to avoid soil erosion and water loss in several civilizations across the world [77,[127], [128], [129], [130], [131], [132], [133]]. Terrace farming is the main method for cultivation in the Mandara Mountains with some original features (Fig. 6) [131,134] compared to other reported terracing systems [132,133]. The main features of terrace farming are: (i) providing ecosystem services, (ii) preserving the biodiversity, and (iii) supporting the agrarian communities [125,132]. Thus, terrace fields are regarded as green infrastructures to mitigate climate change impacts [125,126,132,133]. This is mainly because terrace fields enhance water infiltration (rainfall absorbency) and reduce soil erosion [131,134]. Thus, terrace fields improve land cover and thus moderate extreme temperatures during the dry season, while reducing the flood risks during the rainy season. Summarized, terrace fields contribute to preserve the biodiversity and ecosystems services [125,129].

Etame et al. [61] presented an excellent overview of terrace farming in Djingliya villages within the Mandara Mountains. The Djingliya villages are among the poorest in the Koza subdivision. The poverty is rooted in the largely mountainous topography (undulating lands) with the characteristic poor soil, and the scarcity of arable farms for crop cultivation. The natural scarcity of water coupled to the aforementionned factors justify the reported low agricultural yield, despite great efforts for soil water preservation through indigenous terracing (Fig. 6). The difficulties to maintain the current terracing systems arises from heavy rainfalls. In fact, the Djingliya villages have kept a well-managed system in which the annual surface runoff is very minor, levelling at about 3 % of the early rainfall on the cultivated plots and almost zero on the unplanted plot [61]. Unplanted plot means that infiltration is naturally regulated by land cover within the grass-plant-root system. Under heavy rains, runoff values are greater, increasing to 20 % or more on the cultivated plots. The high values of runoff are the main killers of the terraces, which "may overflow and be washed away if runoff amasses" [61]. The main problem of indigenous terracing in the Mandara Mountains is thus how to avoid such failures and the combined soil erosion (soil loss averaging 4 tons/ha per year)? The logical answer from the presentation above is to avoid the generation of torrents by infiltrating or storing rainfall close to the point of their fall. For this purpose, it is better to have several small terraces than a lower number of bigger ones. Moreover, infiltration and storage pits should be randomly created across the hills, whether the areas are cultivated or not. Another idea, borrowed from the bocage of the Bamiléké, another hilly region in the South of Cameroon [135,136], can consist in supporting some terraces with a green wall, made for example of bamboos [72,137].

### 5.3 Catchment scale RWH

Despite its potential contribution to sustainable water resource management in the Mandara Mountains, previous studies on RWH have been methodically limited, lacking a comprehensive analysis of its contribution to sustainable livelihoods. More so, the discussion of the recent two decades as summurized by Cheo [59] has been partly contraintuitive because the question "to harvest or not to harvest?" is a hindrance for a semi-arid region [18,19]. Clearly, rainwater should be harvested, the only valid question is how best? This study has outlined some issues how starting later, the Mandara Mountains can perform better. The key is to start at the smallest level: a residence (Fig. 4). In this perspective, the catchment area is not comparable to a river basin for the conventional IWRM but is strictly defined by the topography. A catchment area is everything that can be regarded as a stand-alone structure for RWH, this includes individual hills (all the farms and the residences) and standing rocks that are abundant in the region of Kapsiki [138,139].

This presentation suggests that in each catchment area, the best use of water is made. For example, in urban and rural areas, “ensure access to water and sanitation for all” should be the main goal. To achieve that goal, rainwater is harvested within the rural or urban community [113] and completed with the one harvested in the surrundings (e.g. standing rocks, uncultivated areas) [54,63,66,140]. Only when these sources are not sufficient for water supply, people should withdraw groundwater or pipe water from a distand dam, lake or river [18,19]. Clearly, RWH should be mainstreamed in the Mandara Mountains. It is understood that recycling domestic and municipal wastewaters should be considered before water is piped from rivers. The city of Windhoek (Namibia) has elegantly demonstrated that grey water recycling is a powerful tool to mitigate water scarcity [141]. In other words, SDG 6 and its six targets, including achieving universal access to safe drinking water, and reducing the volume of untreated wastewater [142,143], is immediately achievable in the Mandara Mountains while rooting IWRM on RWH. The current approach of IWRM makes a citizen a passive object or at best an active stakeholder in the water management pratice. The presentation herein, on the contrary, is centered on each citizen, on each farm, and on each residence. Water for ecosystem management is considered as well. This corresponds to the old vision of King Parakramabahu (5th century BC): “Let not allow a single drop of water falling as rain flow into the sea without being used for the benefit of mankind” [18,116,118]. Based on this vision, engineering efforts should transform the Mandara hills into networks of drains and reservoirs wherein each drop of rainwater is used and reused times and times before leaving each individual hill catchment [18,19]. The vision of King Parakramabahu has been recently rediscovered and termed as Kilimanjaro Concept [63,64,140].

## 6 Discussion

Integrated water resource management is too often about water allocation, water use and water-related investments among different stakeholders [[144], [145], [146], [147]]. This approach has largely failed to take into account ecosystem needs, societal needs even within a small catchment area. The presentation until now advocates for valuing each drop of rain [18,19,66,[114], [115], [116]]. This approach requires recognition of the full range of direct and indirect benefits (e.g. drinking water, food security) and associated risks (e.g. erosion, flood). The benefits may be cultural, economic, emotional, environmental, social or spiritual [[148], [149], [150], [151], [152], [153], [154], [155], [156], [157]]. This section discusses some paths to valuing water in the Mandara Mountains.

### 6.1 The physics of water distribution

Each drop of water has a mass (e.g. in gram) and is under the influence of gravity. This is the reason why rain falls on earth. Once on the ground, water either: (i) percolates to the subsurface to recharge the aquifer or to replenish groundwater, or (ii) flows to the sea where it becomes saline water. Where there is no geogenic pollution (e.g. As, F, U), groundwater is potable. On the other hand, seawater is not freshwater and shall be desalinized to produce potable water. Rainwater is excellent freshwater and is mostly potable when it is properly collected and stored [30,63,101,102,104]. Clearly, rainwater is the sole water resource that is always freshwater while seawater is always saline. In other words, whether groundwater and surface water are freshwater depends on site-specific considerations like geology, industrialization, or population density [63,102].

Actually, groundwater and surface water are conventionally considered the primary source of freshwater [18,19,158]. This evidence is reflected in the concept of integrated water resource management, wherein rainwater only plays a marginal role, with its significance only preponderant where groundwater and surface water are lacking or are heavilly polluted [64,159]. This approach is counterintuitive for at least two reasons: (i) there are communities that have been relying exclusively on rainwater harvesting for centuries (e.g. Saba in the Dutch Caribbean) [117], and (ii) rainfall is the sole water resource that is evenly distributed at a basin scale. The topography makes the difference and this is why mountains are considered the "water storehouses" or "water towers" for lower laying areas [[160], [161], [162], [163]]. Clearly, at a local scale, the same amount on rain falls on all surfaces, whether the surface is impervious (e.g. rock) or covered by a vegetation. This means that with the progress in civil engineering, the potential exists to store rainfall near to point where it falls [18,19,30,31,148]. Unlike surface and groundwater that are confined in specific locations, rainwater is widely distributed and accessible. All what is needed is a stable ground or favourable geostatics to install harvesting infrastructures. It is surprising to advocate that while this basic principle has been neglected, huge efforts were made to transfer water from dams, lakes and rivers to the points of intensive demands (e.g. cities) [30,31,144,145,164]. However, rational water conservation would have consisted in using rainwater, recycling it, and eventually complete with piped water from distant sources [18,19].

Coming back to the Mandara Mountains, the abscence of perennial rivers, the difficulty to access groundwater, and the shortness of the rainy season makes intensive rainwater infiltration and storage more than a necessity [48,67]. For environmentalists, it can even be considered unethical to let good freshwater flow and get lost as surface runoff [18,19,72]. Accordingly, because the same amount of water falls everywhere, at micro-scale it is easy to realize the concept of "spatial equilibrium state of water resources" [164]. It suffices to scatter storage infrastructures in the whole region. Calculations similar to those made in section 3.3 revealed that more than 300,000 m^3^ of water can be yearly harvested per ha (10000 m^2^) of land, harvesting efficiency of 50 % or larger. It should be recalled that: (i) RWH sustains aquifer recharge, and (ii) the volume of water harvested is no more contributing to flooding and can be used for productive activities on the hills. It is essential to insist on that the cisterns should be almost empty toward the end of the dry season to fullfil their design goals. In other words, apart from tanks for fire fighting, no other tank shall be constantly filled. It is understood that some tanks can be kept filled to bridge particular dry years or for emergencies. However, the current emergency is to supply the Mandara population with safe drinking water [62], and resolve poor land use [61,165].

Tamto-Mamdem et al. [165] recalled that land use in the Mandara Mountains should be revisited for a betterment of food security. Tsozue et al. [166] and Kodji et al. [167] identified erosion by rainwater as the main factor of soil degradation in this region. Overgrazing and the uncontrolled use of fertilizers are other factors responsible for soil degradation [167]. This article insists on the evidence that erosion in the Mandara Mountains results primarily from non-harvested rainwater. Ancient Mandara people were aware of this and solved the problem with their unique and well-researched terrace systems (Fig. 6) [61,87,168]. Now that the terrace system has largely declined, it is essential to harvest and store rainwater where it falls. One main cause of soil errosion is heavy, sudden, and brief rainfall characterized by a large volume of water that cannot percolate into the aquifer nor be properly drained away, causing flash floods and flood pondages [18,19,61,88]. It is evident that this problem is solved if water is harvested and stored locally [18,19]. Past efforts in the Mandara Mountains have overlooked this simple but efficient solution, the lack of an appropriate drainage capacity was regarded as a big problem that cannot be solved without massive investments [88,169].

### 6.2 Hydrological cycle in the Mandara

The processes of water storage as influenced by vegetation coverage is not yet fully understood in mountainous areas in general [84,170]. This deficit of knowledge is the main obstacle to further understanding of hydrological processes and improving water resource management for a better ecosystem in the context of climate change [170].

In the Mandara, it is expected that climate change will have negative impacts on crop and livestock productivity and the overall livelihoods of the people. Changes in temperature and precipitation patterns will mainly result in an unstable water supply in agriculture and land degradation. Changes in life style have led to the partial abandonment of traditional terrace system and water ponding (locally known as biefs) [61,68,81,87]. Therefore, new concepts for a holistic water management are needed. To be successful, such climate-resilient concepts should consider all the threats to the traditional systems and the state-of-the-art knowledge on IWRM [18,19].

It is certain that climate and vegetation changes do affect the hydrological cycle [[170], [171], [172], [173]]. The challenge is to positively influence the water cycle and favour the best vegetation coverage while enabling aquifer recharge. Thus, it is about enabling the availability of soil water in the unsaturated zone. As matter of fact, soil water resulting from rainfall, is partly converted to groundwater (aquifer recharge). In other words, the challenge is to attempt to (i) limit evaporation, (ii) increase infiltration, and (iii) maximize the storage capacities of soils. Etame et al. [61] has already pointed out that this complex system was known to ancient Mandara people while designing their terracing agriculture. The issue is thus to avoid reinventing the wheel. It is about using engineering skills to improve the efficiency of an already efficient system. A key feature in this endeavor will be the proper selection of the vegetation. According to the literature bamboos are one of the best plants for the Mandara Mountains, where acacia is endogeneous. The main advantage of bamboos is that they are fast growing and excellent raw materials for biochar production [[174], [175], [176]]. Biochar can then be used both for water treatment and soil fertility [177,178].

Summarized, Mandara farmers should keep their climate-resilient terracing practices while at the same time improving agrobiodiversity, and introducing crop and plant varieties that can tolerate water and temperature stresses and maximize the availability of water within the unsaturated zone. The presentation above has shown that this task is more successful if each drop of rain is considered. At the term, two main objectives are achieved: (i) universal access to safe drinking water for Mandara people, thus becoming water self-sufficient, and (ii) a better food security with the opportunity to become self-reliant in food supply and even to commercialize the excess.

### 6.3 Ecosystem of the Mandara: Mountain greening amidst climate change

The water demand and the impact of several crops (e.g. maize, millet, sorghum) and plants (e.g. acacia, bamboo, jatropha, sugarcane) on agronomic and environmental factors are well documented and discussed in terms of biomass production, greenhouse gas emissions, nutrient recycling, nutritional value, pest infestation, soil biological attributes, soil conservation, straw recovering for livestock feeding, and weed control [137,[179], [180], [181], [182]]. For some species like bamboo and sugarcane, there is an additional potential to withdraw part of the straw from the fields to produce bioelectricity and cellulosic ethanol [137,182]. Therefore, the challenge for the Mandara consists in selecting the most appropriate species and the most sustainable practices to create a circular economy wherein environmental friendly crops and plants are produced using the best options for water conservation and landscape restoration. This study advocates for rooting the planning on the optimal use of rainwater which is the sole “perennial” source of water on these hills.

From the pure water supply perspective, this study has recalled or re-demonstrated that all three form of floods (e.g. flash floods, flood pondages, river flood) can be largely mitigate if rainfall is locally infiltrated for aquifer recharge or stored in engineered infrastructures for later use. This approach supresses the need for a sophisticated drainage system while making water available for productive activity on the mountains, starting with the restoration of a largely deteriorated landscape. Once the landscape is restored, even heavy, sudden, and brief rainfalls will cause little environmental damage because the infiltration is maximized and the whole Mandara will be filled with small pits and ponds (section 4). Accordingly, it is irresponsible and even unethical not to harvest rainwater [18,19].

Mandara people have long known that their threatened soil is the foundation of their civilization [44,53,131,178,179]. The colonial literature reports on people carrying eroded soil from the valleys back to the hilly terraces [183,184]. This is because fertile soil is vital in converting solar energy into food energy via the photosynthesis process of plants. As a matter of fact, water is needed for these processes and is the most limiting factor in the Mandara hills (Sahel zone) [[44], [45], [46], [47],^61^,^179^]. Soil and water have an immediate impact on agriculture and other primary economic activities, as well as secondary, tertiary, and quartile activities [138,139,[185], [186], [187]]. In the age of global climate change (e.g. changing weather patterns) and increased anthropogenic pressure (e.g. population growth) there is an urgent need to mitigate adverse effects on mountainous landscapes in the Mandara Mountains. Here, these effects are mainly manifested in the form of (i) decreasing soil fertility, (ii) increased deforestation, and (iii) increased soil erosion. While deforestation can be progressively stopped through sensitization and training, soil erosion should be stopped through appropriate rainwater management and soil fertility will be subsequently restored by the biomass from the coming land coverage. Accordingly, in the Mandara hills, there is a need for long-term soil management by controlling afforestation, crop cover, crop rotation, crop selection, soil erosion, soil enrichment, and vegetation cover. As demonstrated herein, this task starts and ends with a proper rainwater management. The goal is best achieved with an "academia for the society" model, rooting progress on heritage.

### 6.4 A new research agenda for the University of Maroua

The Mandara Mountains are key contexts for the sustainable development of Cameroon because of their provision of indispensable goods and services [65]. This recognition was key to the development plans and further actions in these hills since the 1970s [[40], [41], [42], [43], [44]]. Still, Mandara people are among the most disadvantaged in Cameroon, with the highest poverty rates and some of the greatest vulnerability to global climatic change and related risks [64,65,94,188,189]. Worldwide, mountain environments are facing rapid changes in rainfall patterns and temperature [162,190,191]. In Africa in particular, these changes pose challenges for crop production to smallholder farmers [53,54,190,192,193]. Experience has revealed that individual farmers intuitively use multiple adaptation strategies to reduce climate change impacts [53,54,134,190]. The existing challenges and the increasing pressure on Mandara people and resources enforce unsustainable land management practices and land abandonment [134,[188], [189], [190]]. This sad situation calls for holistic strategies to promote sustainable development and to increase the resilience of Mandara populations. Because such a holistic approach is ideally rooted in integrated water resource management (IWRM) (Table 2), interdisciplinary research programs should be established to thinking and accompanying Mandara people into a new era of prosperity. Clearly, it is about opening IWRM to "include energy, food, health and education" [192]. This can be the motivation for interdisciplinary mountain research at the newly created University of Maroua. Presenting this extended call for collaboration is one reason for this article. It is about interdisciplinary mountain research within the University of Maroua and with other domestic and international research groups.

**Table 2:** Perceived order of importance of different water sources in the conventional integrated water resources management (IWRM), and the alternative rooted in rainwater harvesting (RWH). Summ stands for the total amount of water that can be supplied by the conventional IWRM.

Sources	Examples	IWRM	IWRM
		(conventional)	(RWH-based)
**Surface water**	dam, lake, spring	first choice	second choice
**Groundwater**	borehole, well	second choice	third choice
**Rainwater (RWH)**	coutyard, rooftop	third choice	first choice
**Recycled Water**	toilet flushing	fourth choice	fourth choice
**Total**		Summ	harvested rain + Summ

How can Mandara people be universally supplied with safe and reliable basic water services? Answering this question goes beyond estimating the funds needed for construction, operation, and maintenance of needed RWH infrastrucures. It is rather about providing a framework in policy to design interdisciplinary research, capable at enabling such estimations. The immense research need is made clearer when it is considered that the goal is not only water facilities for the supply of the human polulation, much more it is about mountain ecosystems and ecosystem services or more generally about mountain people's wellbeing and livelihoods [173,192,194]. Nsengiyumva [192] highlighted the scarcity of information pertaining on how climate change affects mountain people and their ecosystems in Africa. Jha [190] also insisted on the scarcitz of publications explicitly focuses on adaptation strategies in the African mountains. This means that a decade old efforts to promoting "inclusion of the mountain context in global policy frames for sustainable development and poverty eradication" [161] has not yet reached the Mandara Mountains. Thus, efforts to induce and support the sustainable mountain development agenda to increase the resilience of Mandara populations should be embedded in the existing global agenda.

### 6.5 Feasibility of the concept

This communication is written as a guidance to support Mandara people and institutions involved in their development. The main objective is to increase their capabilities to design and implement small-scale development initiatives rooted in residential and farm scale RWH (Table 2). To this end, the presentation until now has made clear, that RWH is the backborne of all initiatives and shall be supported because it is even a survival issue. The benefits of RWH are numerous but the most important seems to be the in-home water availability, meaning that the many daily journeys to the water sources are suppressed. This is a huge gain of time, particularly for women and children. Table 2 also shows that the amount of rainwater that will enable in-home water is currently not really considered in water allocation in the framework of the conventional IWRM. This section will not discuss the feasibility of universal RWH in any details [[195], [196], [197]], but will be limited on some few financial and technical aspects.

#### 6.5.1 Technical aspects

The technical skills required for the installation of RWH infrastructures can be considered as largely available in the Far North Region of Cameroon. The construction of the main components of such small-scale infrastructures (e.g. cisterns, dams, tanks) requires basic civil engineering skills which are part of the competences acquired at National Advanced School of Engineering at the University of Maroua. It is expected that the University of Maroua will organize special training units, for example in form of certified short courses to train some construction (e.g. skilled masons) and maintenance technicians in remote villages of the Mandara. Similarly, other schools and departments of the University of Maroua will supervise the implementation of the corresponding work packages (e.g. landscape recovering, plant selection, terracing optimization). It should be explicitly stated that the construction of wells and boreholes, and their rehabilitation are not a priority in this endeavor. The focus is on havesting rainfall at the smallest scale and drain it in a gravity driven network [18,19]. This approach excludes the need of energy to pump water (and the related costs).

One essential brake for past projects in these hills has been the lack of skilled personnel [41,45,46,143,198]. It is thus an innovation to present a concept for water supply and landscape restoration that does not need any particularly skilled personnel, including expatriate specialists to be successful. More so, the project can be locally implemented without any governmental support. This corresponds to the science of self-reliance some scientists have been advocating for [26,104,151,[199], [200], [201], [202], [203]]. For example, Josh Kearns has empowered many small communities in Asia and South America to become self-reliant in water supply using biochar-based filtrations systems [[204], [205], [206]].

#### 6.5.2 Financial aspects

**Fig. 7:**
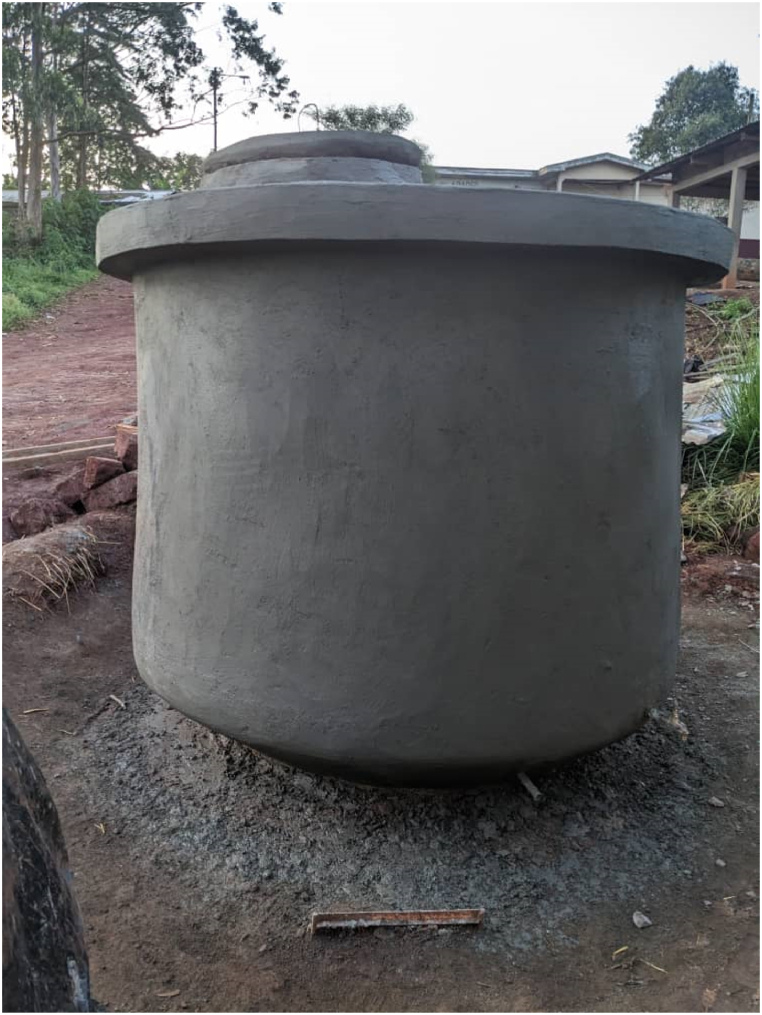
Photograph of an Akkerman Calabash newly constructed in Feutap (Cameroon) (July 2024).

This study strongly advocates for the installation of residential scale RWH infrastructures (e.g. cisterns, gutters, tanks) [[207], [208], [209], [210]]. Accordingly, community level tanks are filled with overflows from individual residential systems [18,19]. Because of the rocky soil, families can use prefabricated containers (e.g. metal or plastic). However, it is better to construct above ground reservoirs like Akkerman Calabashes [104,211] to collect water even from the rondal thatched roofs of traditional houses (Fig. 4). As a matter of fact, new houses should have larger roof catchment areas and tin roofs. For the Mandara Mountains, RWH is not just a water conservation technique; it is a sustainable practice with the capacity to empower local communities to protect their environment, while contributing to the well-being of present and future generations [211,212]. The adoption and promotion of RWH in the Mandara is pivotal in ensuring a sustainable and water-secure future. Over the years, many applicable RWH systems for rural communities have been presented [18,19,[148], [149], [150], [151],^211^,^213^,^214^]. It is difficult to estimate the costs of these systems. Nya and colleagues [116] have recently estimated that a RWH system with a 30 m^3^ burried cistern costs some EUR 3300. For comparison EUR 3300 correspond to about two fly tickets from Berlin (Germany) to Maroua (Cameroun). For individual households the Akkerman's Calabash (Fig. 7) is certainly one of the best choices. The costs in Mokolo is estimated to EUR 350 for 5000 L (5 m^3^). Nevertheless, for a respective catchment and demand an optimal storage size can be estimated by a simple daily water balance model [140,212,215], and installed/constructed, thus assure minimal water loss as overflow.

The population of the Mandara Mountains of Cameroon is 1,467,396 inhabitants (section 3). Assuming a family size of 7 persons [98], 209,628 ″30 m^3^ burried cisterns" are needed for a total cost of EUR 691,772,400 or F CFA 453,608,998,128. The total cost of the World Bank project from 1977 to 1983 was US$ 12.8 million equivalent [45]. At the beginning in 1977, US$1.00 was equal to F CFA 245, in 1983, US$1.00 was equal to F CFA 305, and today, US$1.00 is equal to F CFA 603. These numbers just show that it is difficult to compare the data from then and now. However, while the World Bank project just addressed a part of problem (600 wells, 422 boreholes and 10 dams) the estimation made here addresses the whole population, including people living in their remote villages without any road or electricity. The good news is that, not the whole population need subsidies. As Chétima [131,138,139] has reported, rich Mandara people are building very modern houses in their homes, preferentially on the hill slopes. They just need the information on the importance of RWH insfrastructure to install them in their residence.

## 7 Conclusions

The Mandara Mountains are characterized by significant natural endowments, primarily elevated precipitation levels. The sustainable management of this hydrological asset is crucial for this region. The conservation and efficient utilization of rainwater are paramount, as it remains the sole perennial source of potable and domestic water across the Mandara Mountains’ villages.

The implementation of distributed water storage systems, such as cisterns, small ponds, and tanks, is essential. These distributed reservoirs will serve as principal water storage units, substantially contributing to the stabilization of the regional water ecology. This paper advocates for the development of a novel Mandaran sustainability science framework that not only addresses and interprets emerging environmental challenges but also bolsters societal resilience.

This study provides pivotal information for agronomists, environmental scientists, farmers, policymakers, and other stakeholders to make informed decisions regarding agricultural practices, land use optimization, soil conservation, and rainwater harvesting in the Mandara Mountains. The overarching goal is to promote sustainable agricultural practices and landscape restoration, with concepts and methodologies that are applicable to other mountainous regions globally.

Further, it emphasizes the importance of scholars working on the Mandara Mountains to document and disseminate their research findings widely. This dissemination will create a robust platform to enhance and propel further research into similar hilly regions with high tourism potential. Hence, this paper serves as a clarion call for experts, stakeholders, and scientists to prioritize rainwater harnessing in the Mandara Mountains as an urgent matter for sustainable development.

Increasing awareness regarding the critical role of rainwater through rigorous research and publications will improve the likelihood of its adoption by all societal strata, regardless of economic status. The insights presented herein have the potential to benefit other communities in Africa and Asia with analogous ecological conditions to those of the Mandara Mountains.

## CRediT authorship contribution statement

**Diane Madomguia:** Writing – review & editing, Writing – original draft, Investigation, Conceptualization. **Esther Laurentine Nya:** Writing – review & editing, Writing – original draft, Methodology, Investigation, Conceptualization. **Emma Laureane Njomou-Ngounou:** Writing – review & editing, Writing – original draft, Investigation, Conceptualization. **Nadège Gatcha-Bandjun:** Writing – review & editing, Writing – original draft, Methodology, Conceptualization. **Tulinave Burton Mwamila:** Writing – review & editing, Writing – original draft, Methodology, Conceptualization. **Jules Balna:** Writing – review & editing, Writing – original draft, Methodology, Investigation, Conceptualization. **Emina Halimassia:** Writing – review & editing, Writing – original draft, Methodology, Investigation, Conceptualization. **Jules Metsebo:** Writing – review & editing, Writing – original draft, Methodology, Investigation, Conceptualization. **Wilfried Arsène Letah Nzouebet:** Writing – review & editing, Writing – original draft, Methodology, Conceptualization. **Raoul Rodrigue Tchoumbe:** Writing – review & editing, Writing – original draft, Methodology, Conceptualization. **André Firmin Bon:** Writing – review & editing, Writing – original draft, Methodology, Conceptualization. **Willis Gwenzi:** Writing – review & editing, Writing – original draft, Methodology, Conceptualization. **Chicgoua Noubactep:** Writing – review & editing, Writing – original draft, Supervision, Conceptualization.

## Data availability statement

No new data were created or analyzed in this study. Data sharing is not applicable to this article.

## Funding

This research did not receive any specific grant from funding agencies in the public, commercial, or not-for-profit sectors.

## Declaration of competing interest

The authors declare that they have no known competing financial interests or personal relationships that could have appeared to influence the work reported in this paper.

## References

[1] Kundzewicz Z.W. (1997). Water resources for sustainable development. Hydrol. Sci. J..

[2] Rosegrant M.W. (1997).

[3] Shiklomanov I.A. (2000). Appraisal and assessment of world water resources. Water Int..

[4] Paquin M., Cosgrove C. (2016).

[5] Shah E., Liebrand J., Vos J., Veldwisch G.J., Boelens R. (2018). The UN world water development report 2016, water and jobs: a critical review. Dev. Change.

[6] Boretti A., Rosa L. (2019). Reassessing the projections of the world water development report. NPJ Clean Water.

[7] Domènech L. (2011). Rethinking water management: from centralised to decentralised water supply and sanitation models. Doc. Anal. Geogr..

[8] Younos T. (2011). Paradigm shift: holistic approach for water management in urban environments. Front. Earth Sci..

[9] Hering J.G., Waite T.D., Luthy R.G., Drewes J.E., Sedlak D.L. (2013). A changing framework for urban water systems. Environ. Sci. Technol..

[10] Lo A.G., Gould J., Zhu Q., Gould J., Li Y., Ma C. (2015). Rainwater Harvesting for Agriculture and Water Supply.

[11] Khanal G., Thapa A., Devkota N., Paudel U.R. (2020). A review on harvesting and harnessing rainwater: an alternative strategy to cope with drinking water scarcity. Water Supply.

[12] Umukiza E., Ntole R., Chikavumbwa S.R., Bwambale E., Sibale D., Jeremaih Y., Apollonio C., Petroselli A. (2023). Rainwater harvesting in arid and semi-arid lands of Africa: challenges and opportunities. Acta Sci. Pol. Formatio Circumiectus.

[13] Loper S.A., Zimmerman S.A., Stoughton K.L.M., Pamintuan B.C., Kilgannon E.M. (2024). Geographic information system mapping tool for rainwater harvesting in the United States. J. Water Resour. Plann. Manage..

[14] Teston A., Ghisi E., Martins-Vaz I.C., Scolaro T.P., Severis R.M. (2024). Modular life cycle assessment approach: environmental impact of rainwater harvesting systems in urban water systems. Sci. Tot. Environ..

[15] Gwenzi W., Chaukura N., Noubactep C., Mukome F.N.D. (2017). Biochar-based water treatment systems as a potential low-cost and sustainable technology for clean water provision. J. Environ. Manage..

[16] Duque N., Scholten L., Maurer M. (2024). Exploring transitions of sewer wastewater infrastructure towards decentralisation using the modular model TURN-Sewers. Water Res..

[17] Duque N., Scholten L., Maurer M. (2024). When does infrastructure hybridisation outperform centralised infrastructure paradigms? – Exploring economic and hydraulic impacts of decentralised urban wastewater system expansion. Water Res..

[18] Siphambe T.V., Aliyu A., Souadji K., Bayongwa S.A., Amans T., Fomena-Tchinda H., Banaon P.Y., Gina C.S., Vuai H.A., Farah A.M., Niang A.B., Taicha A., Ahmed S., Bashir A., Abdelbaki C., Mwamila T.B., Gwenzi W., Nya E.L., Noubactep C. (2024). Mitigating flash flooding in the city: drain or harvest?. Water Supply.

[19] Siphambe T.V., Bayongwa S.A., Aliyu A., Amans T., Fomena-Tchinda H., Tchouandem-Nzali C., Mwamila T.B., Nya E.L., Abdelbaki C., Gwenzi W., Noubactep C. (2024). Controlling stormwater at the source: dawn of a new era in integrated water resources management. Appl. Water Sci..

[20] Sample D.J., Liu J. (2014). Optimizing rainwater harvesting systems for the dual purposes of water supply and runoff capture. J. Clean. Prod..

[21] Maurer M. (2022).

[22] Cintura I., Arenas A. (2024). Multi-scale assessment of rainwater harvesting availability across the continental U.S. J. Environ. Manage..

[23] Cook G.C. (2001). Construction of london's victorian sewers: the vital role of Joseph Bazalgette. Postgrad. Med. J..

[24] Younos T., Lee J., Parece T. (2019). Twenty-first century urban water management: the imperative for holistic and cross-disciplinary approach. J. Environ. Stud. Sci..

[25] Younos T., Parece T.E., Lee J., Giovannettone J., Armel A.J. (2021). Introduction to the special issue socio-hydrology: the new paradigm in resilient water management. Hydrology.

[26] Stoler J., Jepson W., Wutich A., Velasco C.A., Thomson P., Staddon C., Westerhoff P. (2022). Modular, adaptive, and decentralised water infrastructure: promises and perils for water justice. Curr. Opin. Environ. Sustain..

[27] Wutich A., Thomson P., Jepson W., Stoler J., Cooperman A.D., Doss-Gollin J., Jantrania A., Mayer A., Nelson-Nuñez J., Walker W.S., Westerhoff P. (2023). MAD water: integrating modular, adaptive, and decentralized approaches for water security in the climate change era. WIREs Water.

[28] Staddon C., Brewis A. (2024). Household water containers: mitigating risks for improved modular, adaptive, and decentralized (MAD) water systems. Water Security.

[29] Prinz D., Pereira L.S. (1996). Sustainability of Irrigated Agriculture.

[30] Herrmann T., Hasse K. (1997). Ways to get water: rainwater utilization or long-distance water supply? A holistic assessment. Water Sci. Technol..

[31] Herrmann T., Schmida U. (2000). Rainwater utilisation in Germany: efficiency, dimensioning, hydraulic and environmental aspects. Urban Water.

[32] Partzsch L. (2009). Smart regulation for water innovation – the case of decentralized rainwater technology. J. Clean. Prod..

[33] van Dijk T. (2019).

[34] van Dijk S., Lounsbury A.W., Hoekstra A.Y., Wang R. (2020). Strategic design and finance of rainwater harvesting to cost-effectively meet large-scale urban water infrastructure needs. Water Res..

[35] Jungraithmayr H., Barbteau D., Seibert U. (1997).

[36] Folifac F., Ndoping Y., Banseka H., Mamba L. (2013). Climate change and water supply adaptation. Lessons from domestic rain water harvesting in Sudano Sahelian Cameroon.

[37] Fonteh M.F. (2013). Water harvesting as an adaptation to climate change in north Cameroon: domestic rainwater harvesting systems. Final Report for Contract # 008/15/GWP-CAf/Core.

[38] Techoro P.S. (2013).

[39] Djaouda M., Nougang M.E., Ldingté J.D., Thélé F.D., Wakayansam R.B., Dawaye D.A., Zébazé-Togouet S.H., Liang S., Nola M. (2023). Public clay pot waters: a hidden risk for diarrheal diseases transmission in a cholera endemic area of Cameroon. Sustain. Water Resour. Manage..

[40] Hoben A. (1976). Margui-Wandala.

[41] World Bank (1977).

[42] Ekobo S., Palmer D., Ripert C. (1978).

[43] Fikry M., Tchala-Abina F. (1978).

[44] Zalla T., Campbell D.J., Holtzman J., Lev L., Trechter D. (1981). Agricultural production potential in the mandara mountains in northern Cameroon. MSU Rural Development Working Paper 17. Department of Agricultural Economics, Michigan State University.

[45] World Bank (1986).

[46] Morel M. (1988).

[47] Laborde J.-P., Morel M. (1991). Aspects climatologiques liés aux possibilités d’alimentation en eau potable par collecte des eaux pluviales dans le Nord-Cameroun. Hydrol. Continent..

[48] Clément D. (1990).

[49] Ripert C., Same-Ekobo A., Enyong P., Palmer D. (1979). Évaluation des répercussions sur les endémies parasitaires (malaria, bilharziose, onchocercose, dracunculose) de la construction de 57 barrages dans les Monts Mandara (Nord-Cameroun). Bulletin de la Societe de Pathologie Exotique et de ses Filiales.

[50] Ripert C.L., Raccurt C.P. (1987). The impact of small dams on parasitic diseases in Cameroon. Parasitol. Today.

[51] Hiol-Hiol F., Mbeyo D.N., Abina F.T., Riej C., Scoones I., Toulmin C. (1996). Sustaining the Soil: Indigenous Soil and Water Conservation in Africa.

[52] Zuiderwijk A.B. (1998).

[53] Nji A., Fonteh M. (2001). Water Harvesting in Western and Central Africa.

[54] Nji A., Fonteh M. (2002). Water harvesting: its potential in the greening and poverty reduction of northern Cameroon. J. Cameroon Acad. Sci..

[55] Seignobos C., Tchotsoua M. (2012). Des stratégies traditionnelles pour la lutte contre l’érosion dans les monts Mandara et dans la plaine du Diamaré. NO du Cameroun. Roose E., Duchaufour H., De Noni G. Lutte antiérosive. réhabilitation des sos tropicaux et protection contre les pluies exceptionnelles. Marseille, IRD Ed., coll. Colloques et séminaires.

[56] Cheo A.E., Voigt H.-J., Mbua R.L. (2013). Vulnerability of water resources in northern Cameroon in the context of climate change. Environ. Earth Sci..

[57] Cheo A.E., Amankwah E., Techoro P.S. (2014). Water harvesting: a potential means for water security in the Far North Region of Cameroon. Agric. Res..

[58] Cheo A.E. (2016). Understanding seasonal trend of rainfall for the better planning of waterharvesting facilities in the Far-North region, Cameroon. Water Utility Journal.

[59] Cheo A.E. (2018).

[60] Bassirou Y., Bitondo D. (2023). Analysis of rainfall dynamics in the three main cities of northern Cameroon. Environ. Monit. Assess..

[61] Etame S.D., Balna J., Deli K.S. (2024). Terracing as an old-style scheme of soil water preservation in Djingliya-Mandara Mountains - Cameroon. Int. J. Innov. Sci. Res. Technol..

[62] Ndjoukap-Kontchou F.M., Oyoa V., Ekoro-Nkoungou H.L., Nlen-wounle B.Y., Mohamadou A. (2024). Contribution of remote sensing and GIS to the location of dam sites for groundwater recharge and flood control: case study in Cameroon (Mandara Mountains). Sustain. Water Resour. Manag..

[63] Marwa J., Lufingo M., Noubactep C., Machunda R. (2018). Defeating fluorosis in the East african rift valley: transforming the Kilimanjaro into a rainwater harvesting park. Sustainability.

[64] Qi Q., Marwa J., Mwamila T.B., Gwenzi W., Noubactep C. (2019). Making rainwater harvesting a key solution for water supply: the universality of the Kilimanjaro concept. Sustainability.

[65] Kammeyer C., Hamilton R., Morrison J. (2020). Averting the global water crisis: three considerations for a new decade of water governance. Georgetown J. Int. Aff..

[66] Huang Z., Nya E.L., Rahman M.A., Mwamila T.B., Cao V., Gwenzi W., Noubactep C. (2021). Integrated water resource management: rethinking the contribution of rainwater harvesting. Sustainability.

[67] Clément D., Jungraithmayr H., Barbteau D., Seibert U. (1997). Man and Water in the Lake Chad Basin.

[68] Djongyang N. (2022). Climate change and some adaptation measures in the Sudano-Sahelian zone of Cameroon. E3S Web of Conferences.

[69] Ishaku H.T., Majid M.R., Johar F. (2012). Rainwater harvesting: an alternative to safe water supply in Nigerian rural communities. Water Resour. Manage..

[70] Kubiku F.N., Nyamadzawo G., Nyamangara J., Mandumbu R. (2022). Effect of contour rainwater-harvesting and integrated nutrient management on sorghum grain yield in semi-arid farming environments of Zimbabwe. Acta Agriculturae Scandinavica B – Soil Plant Sci..

[71] Chiturike P., Nyamadzawo G., Gotosa J., Mandumbu R., Nyakudya I.W., Kubiku F.N.M., Kugedera A.T. (2023). Evaluation of different rainwater harvesting techniques for improved maize productivity in semi‐arid regions of Zimbabwe with sandy soils. J. Sustain. Agric. Environ..

[72] Noubactep C. (2024). Collecte des Eaux Pluviales - Un manuel de sensibilisation. Document Technique préparé pour APADER.

[73] Le Moal M., Gascuel-Odoux C., Ménesguen A., Souchon Y., Étrillard C., Levain A., Moatar F., Pannard A., Souchu P., Lefebvre A., Pinay G. (2019). Eutrophication: a new wine in an old bottle?. Sci. Tot. Environ..

[74] Mycoo M.A., Roopnarine R.R. (2024). Water resource sustainability: challenges, opportunities and research gaps in the English-speaking caribbean small island developing states. PLOS Water.

[75] Fletcher T.D., Andrieu H., Hamel P. (2013). Understanding, management and modelling of urban hydrology and its consequences for receiving waters: a state of the art. Adv. Water Resour..

[76] Fletcher T.D., Shuster W., Hunt W.F., Ashley R., Butler D., Arthur S., Trowsdale S., Barraud S., Semadeni-Davies A., Bertrand-Krajewski J.-L., Mikkelsen P.S., Rivard G., Uhl M., Dagenais D., Mikkelsen P.S. (2015). SUDS, LID, BMPs, WSUD and more – the evolution and application of terminology surrounding urban drainage. Urban Water J..

[77] Zuazo V.H.D., Pleguezuelo C.R.R., Rodríguez B.C., Ruiz B.G., Gordillo S.G., Sacristan P.C., Tavira S.C., García-Tejero I.F., Meena R.S. (2019). Soil Health Restoration and Management.

[78] Burszta-Adamiak E., Spychalski P. (2021). Water savings and reduction of costs through the use of a dual water supply system in a sports facility. Sustain. Cities Soc..

[79] Ortiz S., de Barros Barreto P., Castier M. (2022). Rainwater harvesting for domestic applications: the case of Asunción. Paraguay. Res. Eng..

[80] Mukarram M.M.T., Kafy A.A., Mukarram M.M.T., Rukiya Q.U., Almulhim A.I., Das A., Fattah MdA., Rahman M.T., Chowdhury M.A. (2023). Perception of coastal citizens on the prospect of community-based rainwater harvesting system for sustainable water resource management. Resour. Conserv. Recycl..

[81] David N. (2012). Metals in Mandara Mountains' Society and Culture.

[82] Afamefuna E., Okonkwo E.E. (2019). Exploring the Sukur cultural landscape in Adamawa State of Nigeria: a methodological discussion. Qual. Quant..

[83] Medugu D.W., Umar A.S., Waida J. (2020). Evaluation of wind resource potential in mountainous region: a case study of Mandara Mountains. Int. J. Sci..

[84] Mana D., Konsala S., Adamou I. (2020). Altitudinal distribution of loranthaceae parasites of woody plants on the mandara mountains in the Far North Region, Cameroon. East African scholars. J Agri Life Sci..

[85] BUCREP (2010). Bureau Central des Recensements et des Etudes de Population.

[86] Boutrais J. (1973).

[87] Hallaire A. (1991).

[88] Odunuga S., Adegun O., Raji S.A., Udofia S. (2015). Changes in flood risk in Lower Niger–Benue catchments. Proc. IAHS.

[89] Kana C.E., Nankap Djangue M. (2023). Evaluation of TAMSAT data for rainfall estimates in the northern part of Cameroon. Phys. Environ. Geogr..

[90] Nkiruka E.M., Chinedu A.D., Smart U.N. (2023). Landuse, landcover change dynamics and flooding in the lower Niger basin Onitsha, South Eastern Nigeria. Land Use Pol..

[91] Mainam F. (1999).

[92] Morin S., Seignobos C., Iyébi-Mandjek O. (2000). Atlas de la Province Extrême-Nord Cameroun. Ministère de la Recherche Scientifique et Technique.

[93] David N. (2014). Patterns of slaving and prey–predator interfaces in and around the Mandara Mountains (Nigeria and Cameroon). Africa.

[94] Annavaï N., Wakpounou A., Moussa I. (2021). Les unités Morphostructurales et pédologiques des monts Mandara face à La rétention en eau de surface. Revisita De Geomorfologie.

[95] Seignobos C., Iyebi-Mandjek O. (2000).

[96] Amaah P. (2014). Quantitative and qualitative analysis of the knowledge, attitudes and social representations of cholera in the extreme northern region of Cameroon: the case of Maroua I, Maroua II and Mokolo. Pan Afr. Med. J..

[97] Djaouda M., Mbala E.J., Ewodo M.G., Nziéleu T.G.J., Ombolo A., Zébazé T.S.H. (2018). Assessment of risk factors for cholera outbreaks in Doualaré health area (Maroua, Far North Cameroon). J. Water Environ. Sci..

[98] Zoua W., Djaouda M., Maïworé J., Liang S., Nola M. (2020). Scarcity of potable water and sanitation facilities in the endemic cholera region of North Cameroon. J. Environ. Pollut. Human Health.

[99] Balna J., Gonne B., Madi O.P., Abel T. (2015). Pratiques sylvicoles des pasteurs transhumants dans les agroforêts sèches du Nord Cameroun (Afrique centrale). Int. J. Innov. Appl. Stud..

[100] Balna J., Etame S.D., Hamawa Y., Temgoua K.B., Ganota B., Bernard G., Oumarou P.M., Sali B. (2020). Ethno-botanic study of Tamarindus indica L. in Moutourwa-dry zone of Cameroon. International Journal of Botany Studies.

[101] Davis F. (1891).

[102] Fulton L.V. (2018). A simulation of rainwater harvesting design and demand-side controls for large hospitals. Sustainability.

[103] Khan A.S. (2023). A comparative analysis of rainwater harvesting system and conventional sources of water. Water Resour. Manage..

[104] Akkerman P., Espíndola J.A.G., Flores C.A.C., Pacheco-Vega R., Montes M.R.P. (2020). ‚International Rainwater Catchment Systems Experiences: towards Water Security.

[105] Viviroli D., Dürr H.H., Messerli B., Meybeck M., Weingartner R. (2007). Mountains of the world, water towers for humanity: Typology, mapping, and global significance. Water Resour. Res..

[106] Howard G., Bartram J. (2003).

[107] Stelmach R.D., Clasen T. (2015). Household water quantity and health: a systematic review. Int. J. Environ. Res. Public Health..

[108] Olivry J.C., Hoorelbecke R. (1975). Rapport définitif. Institut de Recherches Hydrologiques, Géologiques et Minières, Onarest/Orstom, Yaoundé, Cameroun.

[109] Ndenecho N.N. (2008). An assessment of the sustainability of water resource development and use in the Chad Basin of Cameroon. J. Cameroon Acad. Sci..

[110] Rana R., Bouri V.A. (2018). Managing water resources in the hilly area of Uttarakhand: strategy and implementation. Sociol Int. J..

[111] Pandey D.N., Gupta A.N., Anderson D.M. (2003). Rainwater harvesting as an adaptation to climate change. Curr. Sci..

[112] Adugna D., Jensen M.B., Lemma B., Gebrie G.S. (2018). Assessing the potential for rooftop rainwater harvesting from large public institutions. Int. J. Environ. Res. Public Health.

[113] Naik P.K., Naik P.K., Prasad G., Mondal K.C. (2024). A design plan for rooftop rainwater harvesting in a large defence establishment in central India. Desalination Water Treat..

[114] Danert K., Motts N. (2009). https://biodiversitylinks.org.

[115] Nguyen X.C., Nguyen T.T.H., Bui X.T., Tran X.V., Tran T.C.P., Hoang T.H., La D.D., Chang S.W., Ngo H.H., Nguyen D.D. (2021). Status of water use and potential of rainwater harvesting for replacing centralized supply system in remote mountainous areas: a case study. Environ. Sci. Pollut. Res..

[116] Nya E.L., Mwamila T.B., Komguem-Poneabo L., Njomou-Ngounou E.L., Fangang-Fanseu J., Tchoumbe R.R., Tepong-Tsindé R., Gwenzi W., Noubactep C. (2023). Integrated water management in mountain communities: the case of Feutap in the Municipality of Bangangté, Cameroon. Water.

[117] Loen S. (2023). Thirsty islands and water inequality: the impact of colonial practices on freshwater challenges in the Dutch Caribbean. Blue Papers.

[118] van Meter K.J., Basu N.B., Tate E., Wyckoff J. (2014). Monsoon harvests: the living legacies of rainwater harvesting systems in South India. Environ. Sci. Technol..

[119] Castejón-Porcel G., Espín-Sánchez D., Ruiz-Álvarez V., García-Marín R., Moreno-Muñoz D. (2018). Runoff water as a resource in the Campo de Cartagena (Region of Murcia): Current possibilities for use and benefits. Water.

[120] Kawata K. (1982). Slow sand filtration for cercarial control in North Cameroon village water supply. Wat. Sci. Tech..

[121] Koop S.H.A., Grison C., Eisenreich S.J., Hofman J., van Leeuwen K. (2022). Integrated water resources management in cities in the world: global solutions. Sustain. Cities Soc..

[122] Lasage R., Aerts J.C.J.H., Mutiso G.C., de Vries A. (2008). Potential for community based adaptation to droughts: sand dams in Kitui, Kenya. Phys. Chem. Earth ABC.

[123] Jadhav M.V., Shaikh E., Gite E., Yadav E. (2012). Sand dam reservoir – need of semi arid areas. Int. J. Eng. Res. Appl..

[124] Negi G.C.S., Samal P.K., Kuniyal J.C., Kothyari B.P., Sharma R.K., Dhyani P.P. (2012). Impact of climate change on the western Himalayan mountain ecosystems: an overview. Trop. Ecol..

[125] Mylona P., Sakellariou M., Giannakopoulos C., Psiloglou B., Kitsara G. (2020). Presented at 9th International Conference on Information and Communication Technologies in Agriculture, Food & Environment (HAICTA 2020), Thessaloniki, Greece, 24–27 September 2020.

[126] Heider K., Rodriguez Lopez J.M., Balbo A.L., Scheffran J. (2021). The state of agricultural landscapes in the Mediterranean: smallholder agriculture and land abandonment in terraced landscapes of the Ricote Valley, southeast Spain. Reg. Environ. Change.

[127] Cicinelli E., Caneva G., Savo V. (2021). A review on management strategies of the terraced agricultural systems and conservation actions to maintain cultural landscapes around the Mediterranean Area. Sustainability.

[128] Sakellariou M., Psiloglou B.E., Giannakopoulos C., Mylona P.V. (2021). Integration of abandoned lands in sustainable agriculture: the case of terraced landscape re-cultivation in Mediterranean Island Conditions. Land.

[129] Chidi C.L., Zhao W., Thapa P., Paudel B., Chaudhary S., Khanal N.R. (2022). Evaluation of traditional rain-fed agricultural terraces for soil erosion control through UAV observation in the middle mountain of Nepal. Appl. Geogr..

[130] Chen L., Wei W., Tong B., Liu Y., Liu Z., Chen S., Chen D. (2024). Long-term terrace change and ecosystem service response in an inland mountain province of China. Catena.

[131] Chétima M. (2011). Par ici l’authenticité! Tourisme et mise en scène du patrimoine culturel dans les monts Mandara du Cameroun. Téoros.

[132] Deng C., Zhang G., Liu Y., Nie X., Li Z., Liu J., Zhu D. (2021). Advantages and disadvantages of terracing: a comprehensive review. Int. Soil Water Conserv. Res..

[133] Jiao W., Yang X., Li Y. (2024). Traditional knowledge's impact on soil and water conservation in mountain agricultural systems: a case study of Shexian Dryland stone terraced System, China. Ecol. Indicat..

[134] Zuiderwijk A.B. (1998). Incorporation and Agricultural Change in the Mandara Mountains of Northern Cameroon.

[135] Tchawa P. (1997). Evolution des techniques traditionnelles de gestion des sols et développement durable: Enseignements tirés de l'étude de deux terroirs bamiléké (Ouest-Cameroun). Les. Cah. d’Outre-Mer.

[136] Tchawa P., Tsayem-Demaze M. (2002). Gestion de l'espace et effets écologiques de l'eucalypcultus en pays Bamiléké (Ouest Cameroun): stratégie paysanne et prise en compte d'un risque perçu. Les. Cah. d’Outre-Mer.

[137] Alamerew A.N., Kozak R., Shrestha A.K., Zhu Z., Wang G. (2024). A way for livelihood improvement: systematic review on bamboo forest research. Trees, Forests and People.

[138] Chétima M. (2019). You are where you build: hierarchy, inequality, and equalitarianism in Mandara Highland architecture. African Studies Rev.

[139] Chétima M. (2023). Traditional and new houses, old and new paths to social status in the Mandara highlands, Cameroon. Can. J. African Stud..

[140] Ndé-Tchoupé A.I., Tepong-Tsindé R., Lufingo M., Pembe-Ali Z., Lugodisha I., Mureth R.I., Nkinda M., Marwa J., Gwenzi W., Mwamila T.B., Rahman M.A., Noubactep C., Njau K.N. (2019). White teeth and healthy skeletons for all: the path to universal fluoride-free drinking water in Tanzania. Water.

[141] Mapani B.S., Shikangalah R.N., Mwetulundila A.L. (2023). A review on water security and management under climate change conditions, Windhoek, Namibia. J. African Earth Sci..

[142] UN SDGs (2015). United nations. Sustainable Development Goals.

[143] Hering J.G., Maag S., Schnoor J.L. (2016). A call for synthesis of water research to achieve the sustainable development goals by 2030. Environ. Sci. Technol..

[144] Biswas A.K. (2004). Integrated water resources management: a reassessment: a water forum contribution. Water international.

[145] Biswas A.K. (2008). Integrated water resources management: is it working?. Int. J. Water Resour. Dev..

[146] Gleick P. (2003). Soft path's solution to 21st-century water needs. Science.

[147] Rijsberman F.R. (2006). Water scarcity: fact or fiction?. Agric. Water Manage..

[148] Hasse R. (1989). Rainwater reservoirs above ground structures for roof catchment. GATE, GTZ) GmbH, Wiesbaden, Germany.

[149] Preul H.C. (1994). Rainfall-runoff water harvesting prospects for greater Amman and Jordan. Water international.

[150] Li F., Cook S., Geballe G.T., Burch W.R. (2000). Rainwater harvesting agriculture: an integrated system for water management on rainfed land in China's semiarid areas. AMBIO: a J. Human Environ..

[151] Cook S. (2004). Assessing the achievements and problems of rural resource management programs in western China: a case study from Gansu Province, China. Environ. Series.

[152] de Melo Branco A., Suassuna J., Vainsencher S.A. (2005). Improving access to water resources through rainwater harvesting as a mitigation measure: the case of the Brazilian semi-arid region. Mitig. Adapt. Strat. Glob. Change.

[153] Varady R.G., Meehan K., Rodda J., McGovern E., Iles-Shih M. (2008). Strengthening global water initiatives. Environment.

[154] Heijnen H. (2013).

[155] Şahin N.İ., Manioğlu G. (2019). Water conservation through rainwater harvesting using different building forms in different climatic regions. Sustain. Cities Soc..

[156] Norman L.M. (2020). Ecosystem services of riparian restoration: a review of rock detention structures in the Madrean Archipelago Ecoregion. Air Soil. Water Res..

[157] Varady R.G., Albrecht T.R., Gerlak A.K., Haverland A.C. (2022). Global water initiatives redux: a fresh look at the world of water. Water.

[158] Domènech L., Saurí D. (2011). A comparative appraisal of the use of rainwater harvesting in single and multi-family buildings of the Metropolitan Area of Barcelona (Spain): social experience, drinking water savings and economic costs. J. Clean. Prod..

[159] Hartigan M. (2009). Help from above: considering rainwater harvesting as an alternative to filtration (innovations case discussion: SONO filters). Innovations.

[160] Barman J., Zuali V.L.H.F., Bindajam A.A., Mallick J., Abdo H.G. (2024). Detection of groundwater conditioning factors in a hilly environment. Appl. Water Sci..

[161] Wehrli A. (2014). Why mountains matter for sustainable development: Switzerland's new mountain program in development cooperation. Mt. Res. Dev..

[162] Wehrli A. (2016). Raise the flag for mountains: enhancing policy dialogue and knowledge sharing through the world mountain forum series. Mountain Res. Dev..

[163] Elmore A.C., Alexiev N., Craig V. (2020). Understanding the world's water towers through high-mountain expeditions and scientific discovery. One Earth.

[164] Wang X., Wang B., Cui F. (2024). Exploring ecosystem services interactions in the dryland: socio-ecological drivers and thresholds for better ecosystem management. Ecol. Indicat..

[165] Tamto-Mamdem E.L., Tsozué D., Matakon E., Apiniel-Atourakail M.R., Moudjie-Noubissie N.M., Basga S.D., Djakba-Basga S., Nzeugang-Nzeukou A., Oyono-Bitom D.L. (2024). Degradation rate/vulnerability potential and fertility status of luvisols in the Mandara Mountains (Far-North Cameroon). Sci. World J..

[166] Tsozue D., Haiwe B.R., Louleo J., Nghonda J.P. (2014). Local initiatives of land rehabilitation in the sudano-sahelian region: case of harde soils in the far north region of Cameroon. Open J. Soil Sci..

[167] Kodji P., Tchobsalaa Ibrahima A. (2021). Impacts of refugees and climate change on agricultural yields in the Sahelian zone of Minawao, Cameroon. Environ. Challenges.

[168] Widgren M. (2010). Besieged palaeonegritics or innovative farmers: historical political ecology of intensive and terraced agriculture in West Africa and Sudan. Afr. Stud..

[169] Odihi J.O. (1996). Urban droughts and floods in Maiduguri: twin hazards of a variable climate. Berichte des Sonderforschungsbereichs.

[170] Zhu G., Yong L., Zhao X., Liu Y., Zhang Z., Xu Y., Sun Z., Sang L., Wang L. (2022). Evaporation, infiltration and storage of soil water in different vegetation zones in the Qilian Mountains: a stable isotope perspective. Hydrol. Earth Syst. Sci..

[171] Song X., Yan C., Xie J., Li S. (2012). Assessment of changes in the area of the water conservation forest in the Qilian Mountains of China's Gansu province, and the effects on water conservation. Environ. Earth Sci..

[172] Sharma H., Ehlers T.A., Glotzbach C., Schmid M., Tielbörger K. (2021). Effect of rock uplift and Milankovitch timescale variations in precipitation and vegetation cover on catchment erosion rates. Earth Surf. Dynam..

[173] Filho W.L., Totin E., Franke J.A., Andrew S.M., Abubakar I.R., Azadi H., Nunn P.D., Ouweneel B., Williams P.A., Simpson N.P. (2022). Understanding responses to climate-related water scarcity in Africa. Sci. Tot. Environ..

[174] Ayer S., Timilsina S., Aryal A., Acharya A.K., Neupane A., Bhatta K.P. (2023). Bamboo forests in Nepal: status, distribution, research trends and contribution to local livelihoods. Adv. Bamboo Sci..

[175] Kenfack-Ananfack G.R., Temgoua E., Avana-Tientcheu M.A. (2023). Farmers' local knowledge of soil fertility in bamboo plantations in the Western Highlands, Cameroon Adv. Bamboo Sci..

[176] González-Prieto Ó., Ortiz Torres L., Vazquez Torres A. (2024). Comparison of waste biomass from Pine, Eucalyptus, and Acacia and the Biochar Elaborated using pyrolysis in a simple double chamber biomass reactor. Appl. Sci..

[177] Gwenzi W., Chaukura N., Noubactep C., Mukome F.N.D. (2017). Biochar-based water treatment systems as a potential low-cost and sustainable technology for clean water provision. J. Environ. Manage..

[178] Fallah N., Pang Z., Lin Z., Lin W., Mbuya S.N., Abubakar A.Y., Kabore M.A.F., Zhang H. (2023). Plant growth and stress-regulating metabolite response to biochar utilization boost crop traits and soil health. Front. Plant Sci..

[179] Holtzman J.S. (1987). http://www.odi.org.uk/pdn/papers/24a.pdf.

[180] Vohland K., Barry B. (2009). A review of in situ rainwater harvesting (RWH) practices modifying landscape functions in African drylands. Agric. Ecosyst. Environ..

[181] Payne D., Spehn E.M., Prescott G.W., Geschke J., Snethlage M.A., Fischer M. (2020). Mountain biodiversity is central to sustainable development in mountains and beyond. One Earth.

[182] de Figueiredo E.B., Jayasundara S., de Oliveira Bordonal R., Rodrigo Panosso A., La Scala Jr N. (2023). Greenhouse gas emissions and offset potential from sugarcane straw for bioenergy production in Brazil. Carbon Footprints.

[183] Denham D. (1826).

[184] Vincent J.F. (1978). Sur les traces du major Denham: le Nord-Cameroun il ya cent cinquante ans. Mandara," Kirdi" et Peul (On the Trail of Major Denham: Northern Cameroon One Hundred and Fifty Years Ago. Mandara, 'Kirdi' and Fulani). Cah. d’Études Afr..

[185] Tiwari P.C., Joshi B. (2012). Environmental changes and sustainable development of water resources in the himalayan headwaters of India. Water Resour. Manage..

[186] Howard C., Flather C.H., Stephens P.A. (2020). A global assessment of the drivers of threatened terrestrial species richness. Nat. Commun..

[187] Mishra A.K., Bhadouria R., Singh S., Tripathi S., Singh P. (2023). Understanding Soils of Mountainous Landscapes.

[188] Iyebi-Mandiek O. (1993). Les migrations saisonnières chez les Mafas, montagnards du Nord-Cameroun: une solution au surpeuplement et un frein à l’émigration définitive. Cah. Sci. Hum..

[189] van Santen J.C. (1998). Islam, gender and urbanisation among the Mafa of north Cameroon: the differing commitment to ‘home’among Muslims and non-Muslims. Africa.

[190] Jha N. (2023).

[191] Wang L., Zhang F., Shi X., Zeng C., Ahmad I., Wang G., Thapa S., Xu X. (2023). Water resources system vulnerability in high mountain areas under climate change. J. Clean. Prod..

[192] Nsengiyumva P. (2019). African mountains in a changing climate: trends, impacts, and adaptation solutions. Mountain Res. Dev..

[193] Carbutt C., Thompson D.I. (2021). Mountain watch: how LT(S)ER is safeguarding Southern Africa's people and biodiversity for a sustainable mountain future. Land.

[194] World Water Council (2023).

[195] Tran S.H., Dang H.T., Dao D.A., Nguyen V.-A., Nguyen L.T., Nguyen V.-A., Han M. (2021). On-site rainwater harvesting and treatment for drinking water supply: assessment of cost and technical issues. Environ. Sci. Pollut. Res..

[196] Raimondi A., Quinn R., Abhijith G.R., Becciu G., Ostfeld A. (2023). Rainwater harvesting and treatment: state of the art and perspectives. Water.

[197] Raimondi A., Quinn R., Gnecco I., Ostfeld A. (2024). New advances in rainwater harvesting and treatment. Water.

[198] Shannon M.A., Bohn P.W., Elimelech M., Georgiadis J.G., Marinas B.J., Mayes A.M. (2008). Science and technology for water purification in the coming decades. Nature.

[199] Noubactep C. (2011). Metallic iron for safe drinking water production. Freiberg Online Geosci..

[200] Kearns J.P. (2016).

[201] Huang Z., Nya E.L., Cao V., Gwenzi W., Rahman M.A., Noubactep C. (2021). Universal access to safe drinking water: escaping the traps of non-frugal technologies. Sustainability.

[202] Bandyopadhyay S. (2024). Small Systems and Emerging Issues.

[203] Noubactep C., Bandyopadhyay S. (2024). Advances in Drinking Water Purification: Small Systems and Emerging Issues.

[204] Kearns J., Flanagan J. (2007). Proceedings of the Third International Conference on Gross National Happiness.

[205] Masten S.J., Harris A., Kearns J., Borrion A., Peters C.A., Gadhamshetty V.R. (2021). Special issue: global environmental engineering for and with historically marginalized communities. Environ. Eng. Sci..

[206] Kearns J., Gropper A., Luis-Muñoz J., Yepéz P. (2023). Adaptable community participatory design to provide water that is Estético, Seguro, y Saludable (pleasant, safe, and healthy) in the Ecuadorian Amazon. Water Security.

[207] Gurung T.R., Sharma A. (2014). Communal rainwater tank systems design and economies of scale. J. Clean. Prod..

[208] Cook S., Sharma A.K., Gurung T.R., Neumann L.E., Moglia M., Chacko P., Sharma A.K., Begbie D., Gardner T. (2019). Rainwater Tank Systems for Urban Water Supply Design.

[209] Karpouzoglou T., Dewulf A., Perez K., Gurung P., Regmi S., Isaeva A., Foggin M., Bastiaensen J., van Hecken G., Zulkafli Z., Mao F., Clark J., Hannah D.M.P.S., Buytaert W., Cieslik K. (2020). From present to future development pathways in fragile mountain landscapes. Environ. Sci. Pol..

[210] Gurung T.R., Sharma A.K., Beal C.D., Stewart R.A. (2023). Sustainable Civil Engineering.

[211] Nthara M., Espíndola J.A.G., Flores C.A.C., Pacheco-Vega R., Montes M.R.P. (2020). International Rainwater Catchment Systems Experiences: towards Water Security.

[212] Nyirenda F., Mhizha A., Gumindoga W., Shumba A. (2021). A GIS-based approach for identifying suitable sites for rainwater harvesting technologies in Kasungu District, Malawi. WaterSA.

[213] Gould E.J. (1995).

[214] Aroka N. (2010).

[215] Pembe-Ali Z., Mwamila T.B., Lufingo M., Gwenzi W., Marwa J., Rwiza M.J., Lugodisha I., Qi Q., Noubactep C. (2021). Application of the Kilimanjaro Concept in reversing seawater intrusion and securing water supply in Zanzibar, Tanzania. Water.

